# Origin of n type properties in single wall carbon nanotube films with anionic surfactants investigated by experimental and theoretical analyses

**DOI:** 10.1038/s41598-021-85248-9

**Published:** 2021-03-11

**Authors:** Susumu Yonezawa, Tomoyuki Chiba, Yuhei Seki, Masayuki Takashiri

**Affiliations:** Department of Materials Science, Tokai University, Hiratsuka, Kanagawa 259-1292 Japan

**Keywords:** Thermoelectrics, Carbon nanotubes and fullerenes, Computational methods

## Abstract

We investigated the origin of n-type thermoelectric properties in single-wall carbon nanotube (SWCNT) films with anionic surfactants via experimental analyses and first-principles calculations. Several types of anionic surfactants were employed to fabricate SWCNT films via drop-casting, followed by heat treatment at various temperatures. In particular, SWCNT films with sodium dodecylbenzene sulfonate (SDBS) surfactant heated to 350 °C exhibited a longer retention period, wherein the n-type Seebeck coefficient lasted for a maximum of 35 days. In x-ray photoelectron spectroscopy, SWCNT films with SDBS surfactant exhibited a larger amount of sodium than oxygen on the SWCNT surface. The electronic band structure and density of states of SWCNTs with oxygen atoms, oxygen molecules, water molecules, sulfur atoms, and sodium atoms were analyzed using first-principles calculations. The calculations showed that sodium atoms and oxygen molecules moved the Fermi level closer to the conduction and valence bands, respectively. The water molecules, oxygen, and sulfur atoms did not affect the Fermi level. Therefore, SWCNT films exhibited n-type thermoelectric properties when the interaction between the sodium atoms and the SWCNTs was larger than that between the oxygen molecules and the SWCNTs.

## Introduction

Single-wall carbon nanotubes (SWCNTs) are novel polyaromatic molecules with small diameters (~ 4 nm) and large length scales (~ 10 μm)^1^. Owing to their unique structure, they exhibit excellent electronic, thermal, and mechanical properties^2–4^. In particular, various electronic characteristics appear depending on the chirality and diameter of the SWCNTs^5,6^. Thus, significant progress has been observed in electronics applications such as thin-film transistors, fuel cells, and lithium-ion batteries^7–9^. Recently, SWCNTs have been considered for application to thermoelectric generators, which produce electricity from ambient thermal energy. SWCNTs exhibit high electrical conductivity and high Seebeck coefficient^10–12^. This suggests that SWCNTs can exhibit good power factor (*PF*) which indicates the generated power per unit temperature difference and is expressed as *PF* = *σS*^2^, where *σ* and *S* are the electrical conductivity and Seebeck coefficient, respectively. For thin-film thermoelectric materials, the *PF* is often used as a thermoelectric performace^13–15^. However, SWCNTs exhibit high thermal conductivity^16^. Thus, SWCNTs have a detrimental effect on the dimensionless figure of merit (*ZT*) which indicates the energy conversion efficiency of materials and is expressed as *ZT* = *σS*^2^*T*/*κ*, where *κ* is the thermal conductivity. In addition to their good thermoelectric performance, SWCNTs are inherently flexible; hence, they can be used to create flexible thermoelectric generators with high thermoelectric performance^17–21^. Therefore, a flexible thermoelectric generator using SWCNTs can be used as a power supply for wearable electronic devices and wireless sensor networks^22–25^.

One of the most important aspects to consider in the application of SWCNTs to thermoelectric materials is an understanding of the n-type semiconducting properties of SWCNTs. Owing to defects, pristine SWCNTs exhibit n-type properties, which immediately change to p-type ones when the SWCNTs are exposed to air^26^. Under these circumstances, many researchers have attempted to fabricate SWCNTs with n-type properties using various experimental approaches^27–31^. Nakashima et al. prepared air-stable n-type SWCNTs doped with benzimidazole derivatives and discussed their air-stable mechanism^32^. Nonoguchi et al. investigated new doping reagents of ordinary salts with crown ethers to create air- and thermally stable n-type SWCNTs^33^.

These pioneering studies motivated us to explore air-stable n-type SWCNTs using a facile fabrication process. Thus, we prepared SWCNT films with different anionic surfactants followed by heat treatment^34^. Anionic surfactants are the most common type of surfactants and have excellent salt resistance and solubility in organic solvents. We used sodium dodecyl sulfate (SDS), sodium dodecylbenzene sulfonate (SDBS), and sodium cholate (SC), and found that the SWCNT films with SDBS exhibited the most air-stable n-type properties. The next step was to analyze the structure of n-type SWCNTs in detail, and to computationally clarify the origin of the n-type property of SWCNTs with anionic surfactants.

In this study, we explored the origin of n-type SWCNT films with anionic surfactants using experimental analyses and first-principles calculations. The n-type SWCNT films were prepared by adding different anionic surfactants, followed by heat treatment. The structural and thermoelectric properties excluding the thermal conductivity of the SWCNT films prepared using various surfactants were analyzed. The thermal conductivity will be measured in the future. The electronic band structure and density of states (DOS) of the SWCNT films with varying surfactants were determined from first-principles calculations.

## Experimental setup

Super growth-carbon nanotubes (SG-CNTs; ZEONANO SG101, purity > 99%, ZEON) were used as the SWCNTs. The anionic surfactants used in this study were SDS: NaC_12_H_25_SO_4_ (purity > 95%, Fujifilm Wako Pure Chemicals), SDBS: C_18_H_29_NaO_3_S (purity > 95%, Tokyo Chemical Industry), and SC: C_24_H_39_NaO (purity > 98.5%, Fujifilm Wako Pure Chemicals). These surfactants were used in their received state without further purification. The SWCNT dispersion solution was prepared by dispersing SWCNT powders in deionized water. The concentration of SWCNTs in the dispersion solution was maintained at 0.2 wt%, and the weight of the added surfactant was 5 times that of the SWCNT powders. To disperse the SWCNTs and surfactant more uniformly, an ultrasonic homogenizer (SONICS85: AZ-1 Corporation) was used at an output power of 60 W for 30 min.

The SWCNT films with surfactants were formed by drop-casting, followed by heat treatment at different temperatures. To form a film over the entire area, 0.9 mL of SWCNT dispersion solution was dropped on a glass substrate (25 mm × 20 mm × t1.1 mm). After the films were dried in air for 24 h, they were heat-treated in an electric furnace to remove water from the surfactant and connect the SWCNTs and elements of the surfactants. The furnace was filled with a gas mixture of Ar (95%) + H_2_ (5%) at atmospheric pressure, and the treatment temperature was varied from 150 to 450 °C, while the treatment time was maintained at 1 h. The approximate film thickness was 10 μm.

The in-plane Seebeck coefficients, *S*, of the SWCNT films were measured at approximately 293 K with an accuracy of ± 5% using a system developed in-house. To evaluate the time dependence of the Seebeck coefficient, the measurement was first performed at 1-day intervals for a total of 7 days. The detailed measurement procedure is described in our previous report^19^. In brief, one end of the film was placed on a heat sink, whereas the other end was placed on a heater. Two K-type thermocouples (diameter of 0.1 mm) were held at the middle of the film with a gap of 13 mm between them. The temperature difference between the thermocouples was controlled from 1 to 5 K, whereas the thermoelectric power was measured at 1 K increments. The Seebeck coefficient, *S*, was obtained from the linear approximation of the voltage–temperature slope.

The chemical structures of the SWCNTs were evaluated using a Raman microscope with an Ar^+^ laser beam excitation at 514.5 nm (XploRA: HORIBA) and X-ray photoelectron spectroscopy (XPS; ULVAC-PHI Quantum 2000). The microstructures of the SWCNT films with surfactants were characterized by transmission electron microscopy (TEM) (JEM-2100F, JEOL). The color mapping of the atomic distribution was evaluated using an energy dispersive X-ray analyzer (EDX) in a JEM-2100F.

### Calculation details

Based on the density functional theory, the electronic band structure and DOS of the SWCNT films with surfactants were calculated using Quantum ESPRESSO software version 5.2^35,36^. To simplify the calculation, we employed the semiconducting type of SWCNTs while both metal and semiconducting types of SWCNTs were formed during the synthesis. This is because the presence of the metallic SWCNTs does not vary significantly the influence of the molecules on top of the nanotubes^37^. In the semiconducting type of SWCNTs, an SWCNT with chirality of (8,4) and a diameter of 0.84 nm was chosen to compare the calculation analyses with experimental results. This is because the chirality of (8,4) was known to exhibit semiconducting properties corresponding to that of the SG-CNTs^38,39^, and the diameter of 0.84 nm largely coincided with the experimental result. The initial geometry of the SWCNTs was obtained from the TUBEGEN 3.4 program^40^.

To make the model systems, pristine SWCNTs and SWCNTs interacting with oxygen atoms or molecules, water molecules, or sodium or sulfur atoms were prepared. The adsorption was performed with no replacement of the carbon atoms, and the positions of the adsorption atoms were determined after structural optimization considering the Van der Waals interactions between SWCNTs and atoms/molecules placed on top using the DFT-D3 method^41^. To avoid interaction between SWCNTs, each SWCNT was placed in the center of a supercell, and vacuum layers of 1.0 nm were provided on the top, bottom, left, and right of each SWCNT.

Calculations were performed based on ultrasoft pseudopotentials and Perdew–Burke–Ernzerhof generalized gradient approximation for the exchange correlation functional^42,43^. The Monkhorst–Pack method was used for sampling K meshes. Grids with dimensions of 1 × 1 × 6 were used for self-consistent field (SCF) calculations, whereas grids with dimensions of 1 × 1 × 40 were used for non-self-consistent field (NSCF) calculations. The energy cutoffs and electron charge densities were set to 66 and 326 Ry, respectively. The coordinates of the carbon atoms in the SWCNTs and the lattice constant were fixed, and the structure was optimized at the coordinates of the surfactant atoms.

## Results

### Experimental results

**Figure 1:**
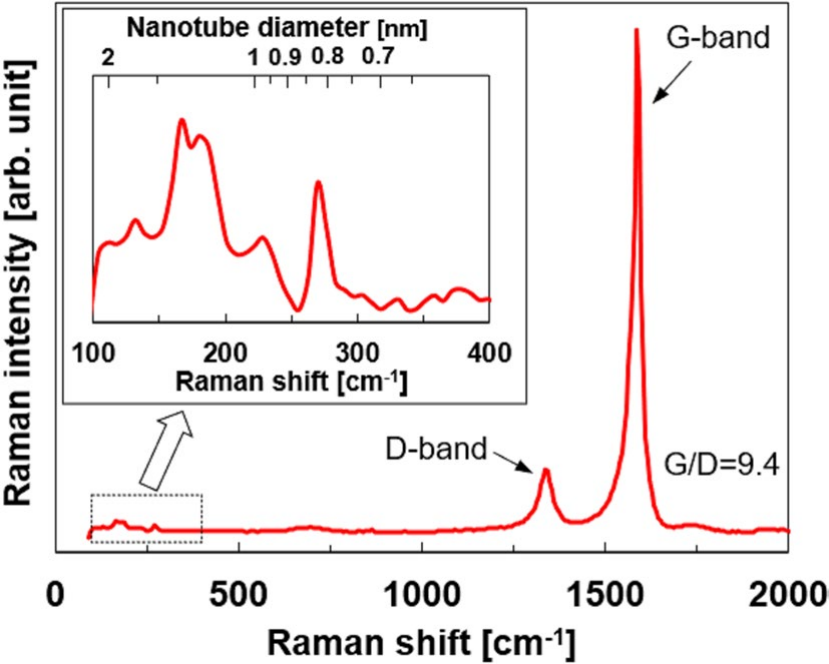
Raman spectrum of SWCNTs (SG-CNTs). Inset indicates a detailed analysis of the RBM modes ranging from 100 to 400 cm^–1^.

The Raman spectrum of the SWCNTs is shown in Fig. 1. Radial breathing mode (RBM) peaks were observed in the range 100–300 cm^−1^, as shown in the inset of Fig. 1. There were several RBM peaks in the spectrum, indicating that they exhibited a chirality distribution and different diameters. We calculated the SWCNT diameter (*d*) using the RBM frequency (*ν*), which is expressed as *d* (nm) = 223.75/*ν* (cm^−1^)^44^. As a result, the SWCNT diameters ranged from 0.8 to 1.5 nm. In particular, distinct peaks appeared at 166.5, 180.3, and 269.6 cm^–1^, corresponding to diameters of 1.34, 1.24, and 0.83 nm, respectively. Conversely, the high-frequency region of the Raman spectrum of the SWCNTs revealed G- and D-bands at approximately 1590 and 1350 cm^−1^, respectively. In general, the G-band is a graphite-derived spectrum of carbon atoms in a hexagonal lattice, whereas the D-band appears when the defects of the carbon basal plane lattices are included in the crystal lattice of SWCNTs^45^. Thus, the ratio (*I*_G_/*I*_D_) between the integral intensities of the G- and D-bands reflects the defect density. The *I*_*G*_/*I*_*D*_ ratio for the SWCNTs was 9.4, which was lower than that of SWCNTs synthesized using other methods^46,47^.

**Figure 2:**
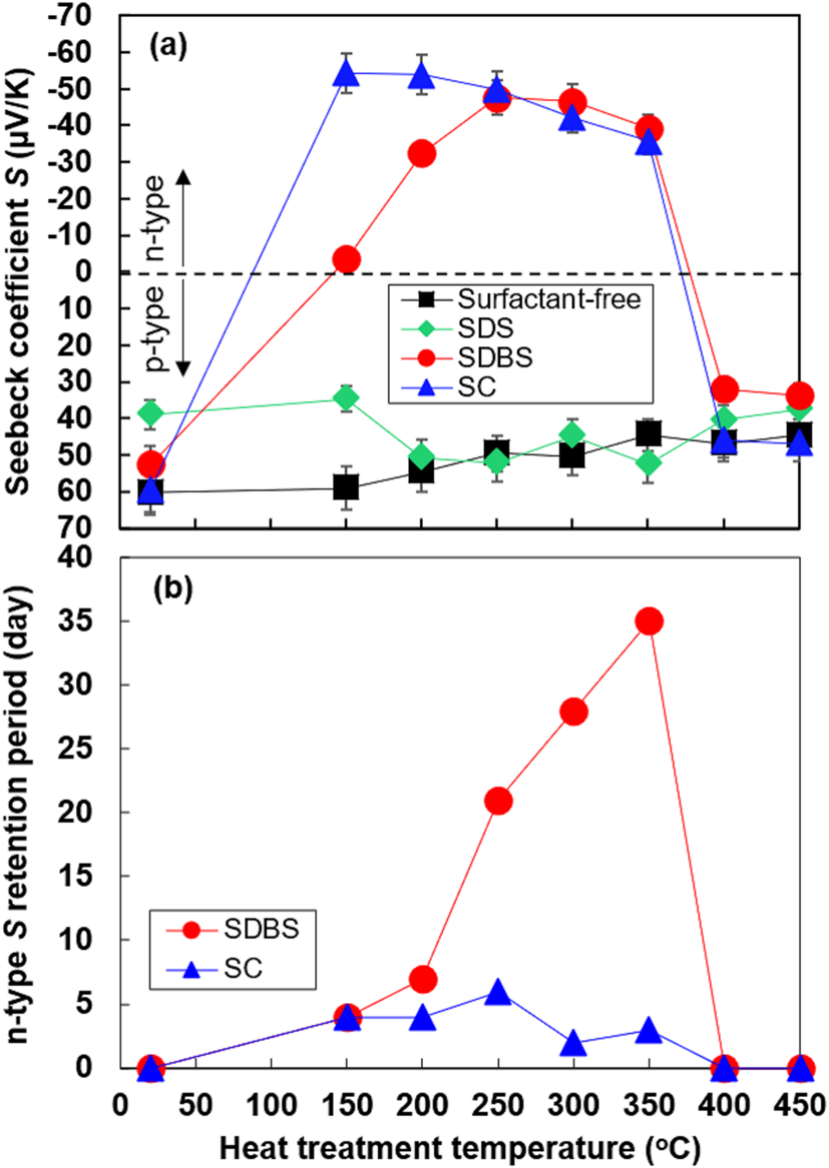
(**a**) Seebeck coefficients of SWCNT films with different surfactants as a function of heat-treatment temperature. (**b**) Retention period of n-type Seebeck coefficients of the selected SWCNT films depending on the heat-treatment temperature.

The relationship between the in-plane Seebeck coefficient of the SWCNT films with different surfactants and the heat treatment temperatures is shown in Fig. 2a ^34^. All the Seebeck coefficients were measured within 1 day of heat treatment. The Seebeck coefficients of the surfactant-free SWCNT films and SWCNT films with SDS exhibited positive (p-type) Seebeck coefficients at all heat treatment temperatures. Conversely, the SWCNT films with SDBS and SC exhibited negative (n-type) Seebeck coefficients when the heat treatment was performed in the range 150–350 °C. The negatively highest Seebeck coefficient for both SWCNT films with SDBS and SC was approximately − 50 μV/K. To investigate the stability of the n-type Seebeck coefficient of the SWCNT films, the retention period of the n-type Seebeck coefficient as a function of the heat-treatment temperature is shown in Fig. 2b. The n-type Seebeck coefficient of the SWCNT films with SDBS showed relatively high stability. The stability improved drastically when the heat treatment temperature was approximately 200 °C. In particular, the maximum retention period was 35 days at a treatment temperature of 350 °C. Conversely, the stability of the n-type Seebeck coefficient of SWCNT films with SC was lower than that of the SWCNT films with SDBS. The maximum retention period was 6 days at a treatment temperature of 250 °C.

**Figure 3:**
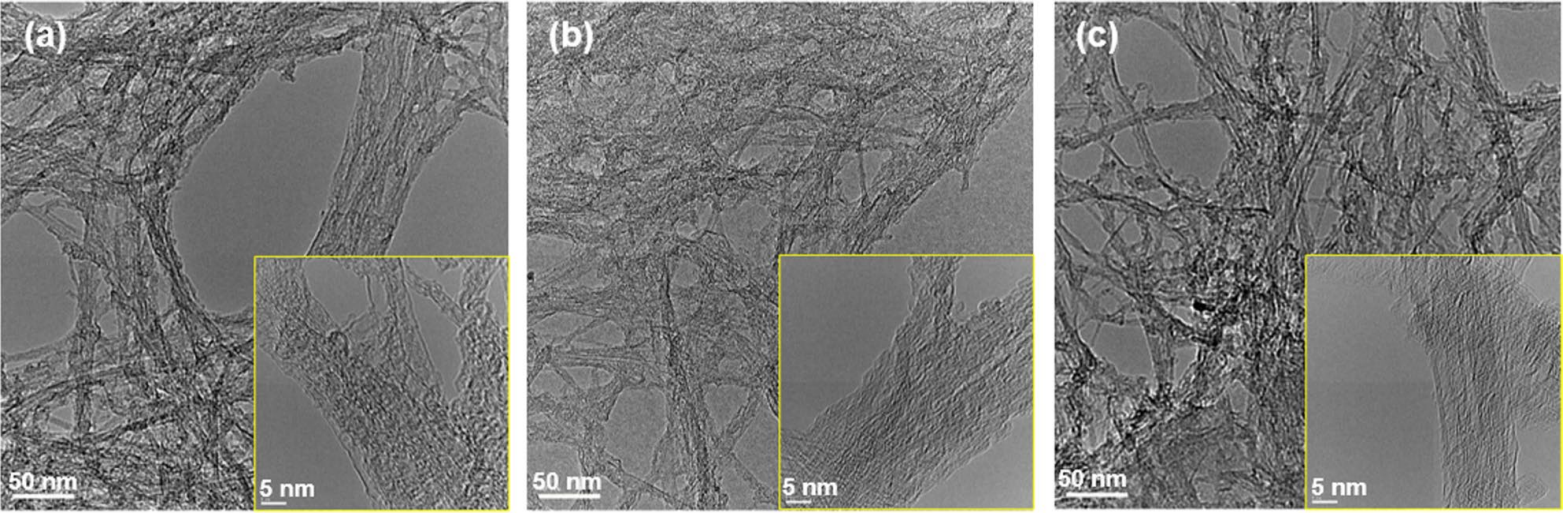
Surface TEM images of SWCNT films with different surfactants at heat treatment temperatures exhibiting long retention period of an n-type Seebeck coefficient. (**a**) SDS at 150 °C, (**b**) SDBS at 350 °C, and (**c**) SC at 250 °C.

Figure 3 shows TEM images of the SWCNT films with different surfactants. In particular, as the stability of the n-type Seebeck coefficient of the SWCNT films depended on the heat treatment temperature, the typical TEM images of the SWCNT films for each surfactant at the temperature allowing for the longest retention period are presented; SDS at 150 °C, SDBS at 350 °C, and SC at 250 °C. The insets of the figures show that dozens of SWCNTs were bundled. No matter which surfactant was used, the elements of the surfactant were attached around the SWCNTs, and, thus, no difference was observed between the different surfactants.

**Figure 4:**
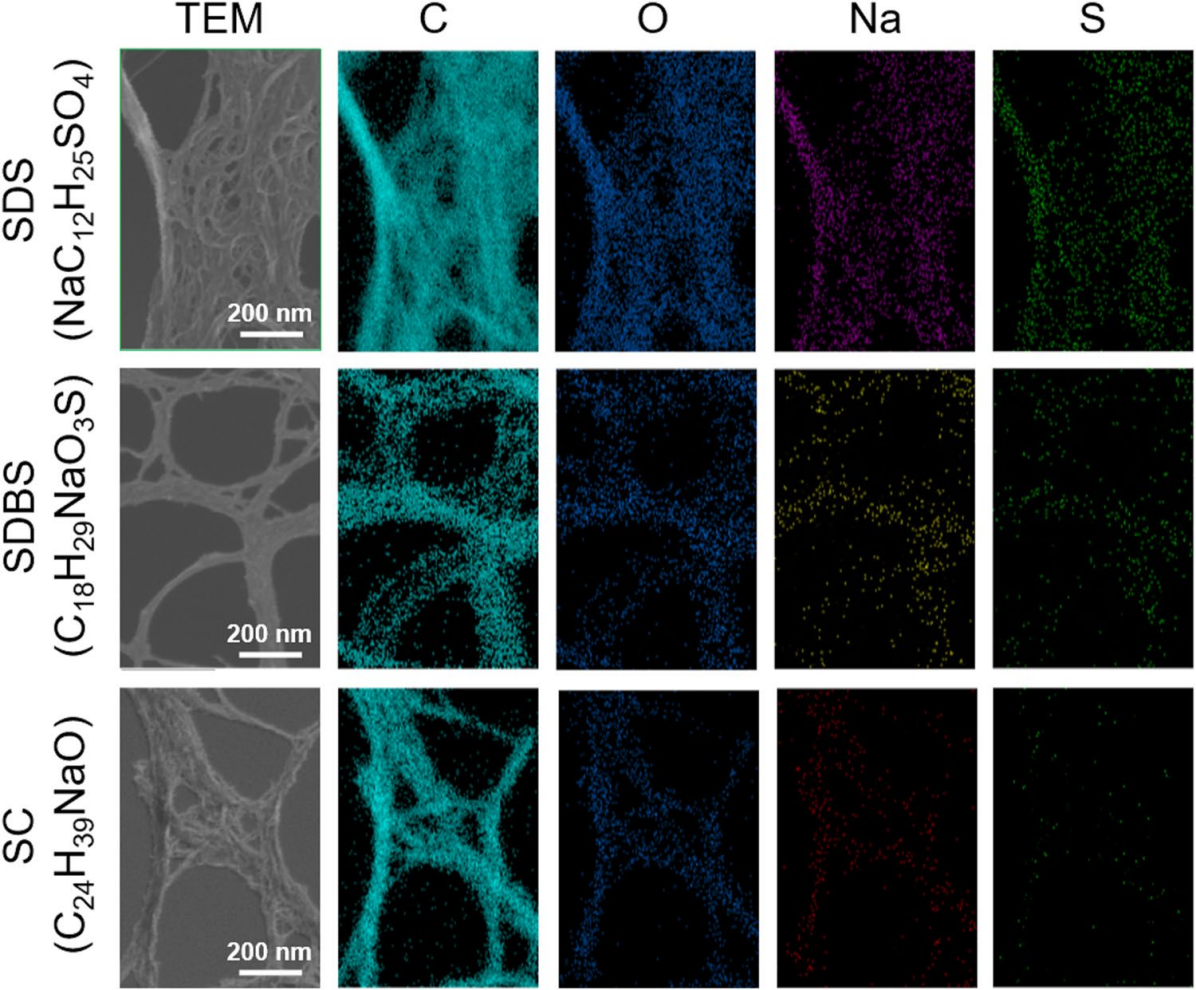
Color mapping of atomic distribution in SWCNT films with different surfactants, determined by EDX.

The color mapping of the atomic distribution in the SWCNT films with different surfactants is shown in Fig. 4. Color mapping and TEM observations were performed simultaneously using the same samples. In the SWCNT film with SDS (NaC_12_H_25_SO_4_), a signal from oxygen was clearly observed. The brightness of the signals originating from the sodium and sulfur atoms was lower than that of the signals originating from the oxygen ones, indicating that many oxygen atoms or molecules or water molecules were adsorbed on the SWCNT surface. In the SWCNT film with SDBS (C_18_H_29_NaO_3_S), the brightness of the signal from oxygen decreased, and that from sodium increased, compared to the SWCNT film with SDS. In the SWCNT film with SC (C_24_H_39_NaO), the signal from the oxygen was the strongest, followed by that from sodium. A negligible signal from sulfur was detected because the SC did not contain sulfur atoms.

**Figure 5:**
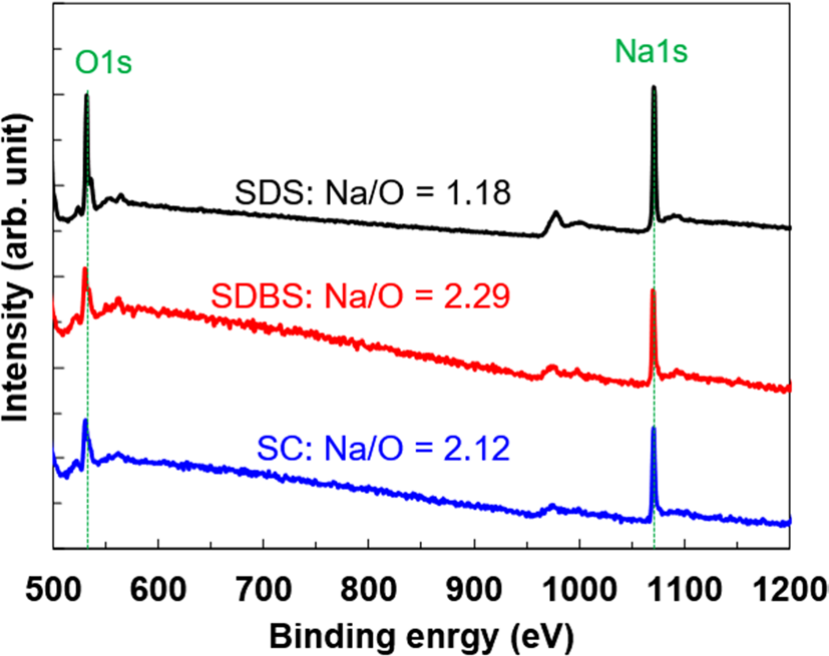
XPS spectra of SWCNT films with different surfactants, determined by XPS.

The color mapping using EDX is conveniently to estimate the atomic distribution on the surface, but it is difficult to perform the quantitative analysis. Therefore, to clarify the quantitative balance between oxygen and sodium on the SWCNTs, the XPS analysis was performed, as shown in Fig. 5. In all the SWCNT films with different surfactants, the spectra of O1s and Na1s were observed in the range of binding energy from 500 to 1200 eV. The highest peak intensity ratio of Na/O was 2.29 at the SWCNT film with SDBS while the lowest one was 1.18 at the SWCNT film with SDS. These results indicated that a relatively large amount of sodium atoms existed on the surface of the SWCNT film with SDBS compared to those of the SWCNT films with SDS and SC. Therefore, we concluded that the SDBS surfactant had sufficiently covered the SWCNTs and prevented oxygen atoms or molecules and water molecules from adhering to the SWCNT surface.

### Calculation analysis

**Figure 6:**
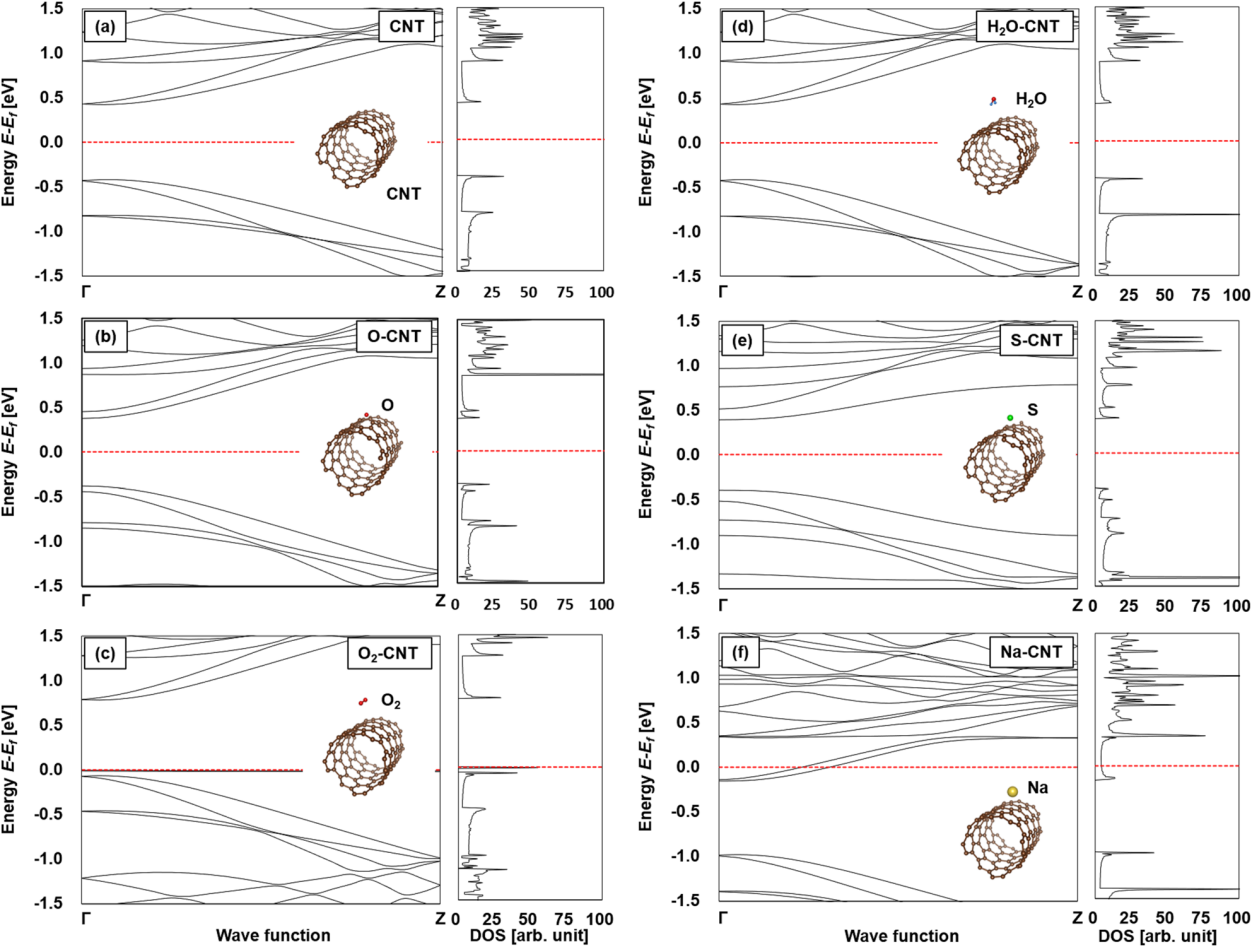
Calculated electronic-band diagrams and densities of states (DOSs) of (**a**) pristine SWCNT and SWCNTs with (**b**) oxygen atom, (**c**) oxygen molecule, (**d**) water molecule, (**e**) sulfur atom, or (**f**) sodium atom attached.

Figure 6 shows the electronic band structure and DOS of the pristine SWCNTs and the SWCNTs surrounding various atoms. The insets describe the molecule models showing the positional relationship between the SWCNTs and surrounding atoms. The diameter of the SWCNTs was set at 0.84 nm, as mentioned in the section of experimental setup, even though there were three distinct peaks observed in the Raman spectra, as shown in the inset of Fig. 1. This is because 0.84 nm is the smallest diameter among the three peaks, corresponding to the minimum atomic number in a unit cell, which contributes to making calculations as short as possible. As a result of several patterns of structural optimization, adjusting the initial positions of the added atoms or molecules, it was found that the stable position of the atoms or molecules was different for each type of atom or molecule. The oxygen atom was located just above the carbon bonds at a distance of 0.093 nm. The oxygen molecule was located away from the SWCNT surface compared to the oxygen atom, at a distance of 0.331 nm. The water molecule was located farther away from the SWCNT surface compared to the oxygen molecule, at a distance of 0.365 nm. The sulfur atom was located just above the carbon bonds at a distance of 0.166 nm, whereas the sodium atom was located directly above the center of the six-membered ring at a distance of 0.222 nm.

When no atoms were on the SWCNT (i.e., the pristine SWCNT), the Fermi level was exhibited approximately in the middle of the bandgap (Fig. 6a). The Fermi level did not shift to the side of the conduction band because the calculations in this study did not consider the defects in the SWCNTs. When the oxygen atom was on the SWCNT, the Fermi level did not shift from that observed for the pristine SWCNT (Fig. 6b). In the case of oxygen molecules, the Fermi level shifted considerably to the edge of the valence band, as electrons were transferred from the SWCNT to the oxygen molecules, indicating a positive (p-type) Seebeck coefficient (Fig. 6c). When the water molecule was on the SWCNT, the Fermi level was exhibited approximately in the middle of the bandgap (Fig. 6d), which was the same trend observed as when the sulfur atom was on the SWCNT (Fig. 6e). Conversely, when the sodium atom is located above the SWCNT, the Fermi level shifts upwards and overlaps with the conduction band, as electrons are transferred from the sodium atom to the SWCNT, indicating a negative (n-type) Seebeck coefficient (Fig. 6f). Therefore, we concluded that the oxygen molecules caused a positive Seebeck coefficient, and the sodium atoms caused a negative Seebeck coefficient. Moreover, the distance between SWCNT and sodium atom (0.222 nm) is shorter than that between SWCNT and oxygen molecule (0.331 nm). This indicated that sodium atom has a more dominant influence of Fermi level shift than oxygen molecule.

Here, it should be noted that we could not perform the calculations using the SWCNT systems with diameters larger than 1.24 and 1.34 nm, corresponding to the experimental results shown in the inset of Fig. 1. This is because the number of atoms in a unit cell became large when the diameter of the SWCNTs increased^36^, resulting in the requirement of a computational capacity beyond the capacity of our system. However, although the magnitude of the Seebeck coefficient is known to depend on the diameter of the SWCNTs, we consider that the appearance of p-type or n-type SWCNTs with surfactants that possess larger diameters is the same when the diameter of the SWCNTs is small^48^. This is because the transfer of electrons between the oxygen molecules or sodium atoms and the SWCNTs does not depend on the diameter of the SWCNTs; that is, there is no significant change in the band structure and DOS of SWCNTs with larger diameters.

## Discussion

We studied and described how the structure and elements of the surfactants contribute to the n-type thermoelectric properties of the SWCNTs. The coverage of the surfactant over the SWCNTs differs depending on the type of surfactant used. Using simulations, Hirano et al. reported the superiority of SDBS for CNT surface coverage compared to several other surfactants^49^. In this study, when the surfactant dispersed the SWCNTs, the surfactant worked to envelop the entire SWCNT; the coating condition was thought to inhibit the oxidation of the SWCNTs. The coverage advantage is enhanced when the surfactants have chains and benzene rings, such as in SDBS. Thus, when the SWCNTs covered with SDBS surfactant are deoxidized by heat treatment, the sodium atoms introduced by the surfactant become more numerous. At this time, the SWCNTs exhibited an n-type Seebeck coefficient. However, the Seebeck coefficient changed to p-type after a long period of time (35 days) because the oxygen molecules adsorbed to the SWCNT surface again. Conversely, when SDS and SC were used as surfactants, the coverage of the SWCNT surface was insufficient, allowing the oxygen molecules to penetrate the SWCNT surface. In this case, the Seebeck coefficient either did not change to n-type or had an unstable n-type state. Therefore, we clarified the origin of n-type thermoelectric properties in the SWCNT films with anionic surfactants and the cause of the deterioration of n-type properties over time. To further stabilize the n-type properties for a long time, it is necessary to use a sodium-based surfactant that tightly binds to the SWCNT surface or seal the surface to prevent oxygen molecules from entering the SWCNT.

## Conclusions

We investigated the effect of surfactants on the thermoelectric properties of SWCNT films at different heat treatment temperatures. Pristine SWCNTs and SWCNT films with SDS surfactant maintained their p-type properties in all temperature ranges. The SWCNTs with SDBS and SC surfactants showed n-type properties when subjected to heat treatment at temperatures ranging from 150 to 350 °C. In particular, the SWCNTs with SDBS exhibited a longer retention period in the n-type property, with a maximum of 35 days at 350 °C. The electronic band structure and DOS of the SWCNTs (chirality (8,4)) with oxygen atoms, oxygen molecules, water molecules, sulfur atoms, and sodium atoms were evaluated using first-principles calculations. It was found that sodium atoms and oxygen molecules moved the Fermi level closer to the conduction and valence bands, respectively, resulting in a change in the Seebeck coefficient. Therefore, we demonstrated that the structure and elements of the surfactants were responsible for the presence and stability of the SWCNT film’s n-type property.

## Data Availability

The data that support the findings of this study are available from the corresponding author upon reasonable request.

## References

[1] Iijima S, Ichihashi T (1993). Single-shell carbon nanotubes of 1-nm diameter. Nature.

[2] De Volder MFL, Tawfick SH, Baughman RH, Hart AJ (2013). Carbon nanotubes: present and future commercial applications. Science.

[3] Shiomi J, Maruyama S (2006). Heat conduction of single-walled carbon nanotube isotope superlattice structures: a molecular dynamics study. Phys. Rev. B.

[4] Yu MF, Files BS, Arepalli S, Ruoff RS (2000). Tensile loading of ropes of single wall carbon nanotubes and their mechanical properties. Phys. Rev. Lett..

[5] Odom TW, Huang JL, Kim P, Lieber CM (1998). Atomic structure and electronic properties of single-walled carbon nanotubes. Nature.

[6] Kiang C-H, Endo M, Ajayan PM, Dresselhaus G, Dresselhaus MS (1998). Size effects in carbon nanotubes. Phys. Rev. Lett..

[7] Okimoto H (2010). Tunable carbon nanotube thin-film transistors produced exclusively via inkjet printing. Adv. Mater..

[8] Girishkumar G (2005). Single-wall carbon nanotube-based proton exchange membrane assembly for hydrogen fuel cells. Langmuir.

[9] Wu H, Meng Q, Yang Q, Zhang M, Wei Z (2015). Large-area polyimide/SWCNT nanocable cathode for flexible lithium-ion batteries. Adv. Mater..

[10] Vavro J (2003). Thermoelectric power of p-doped single-wall carbon nanotubes and the role of phonon drag. Phys. Rev. Lett..

[11] Meng C, Liu C, Fan S (2010). A promising approach to enhanced thermoelectric properties using carbon nanotube networks. Adv. Mater..

[12] Small JP, Perez KM, Kim P (2003). Modulation of thermoelectric power of individual carbon nanotubes. Phys. Rev. Lett..

[13] Lee W (2016). Improving the thermoelectric power factor of CNT/PEDOT:PSS nanocomposite films by ethylene glycol treatment. RSC Adv..

[14] Hosokawa Y (2018). Thermal annealing effect on structural and thermoelectric properties of hexagonal Bi_2_Te_3_ nanoplate thin films by drop-casting technique. Jpn. J. Appl. Phys..

[15] Singh S (2020). High Seebeck coefficient in thermally evaporated Sb-In co-alloyed bismuth telluride thin film. J. Appl. Phys..

[16] Fujii M (2005). Measuring the thermal conductivity of a single carbon nanotube. Phys. Rev. Lett..

[17] Yamamuro H (2018). Combination of electrodeposition and transfer processes for flexible thin-film thermoelectric generators. Coatings.

[18] Kato K (2014). Flexible porous bismuth telluride thin films with enhanced figure of merit using micro-phase separation of block copolymer. Adv. Mater. Interfaces.

[19] Kobayashi A, Konagaya R, Tanaka S, Takashiri M (2020). Optimized structure of tubular thermoelectric generators using n-type Bi_2_Te_3_ and p-type Sb_2_Te_3_ thin films on flexible substrate for energy harvesting. Sensors Actuators A.

[20] Khumtong T, Sukwisute P, Sakulkalavek A, Sakdanuphab R (2017). Microstructure and electrical properties of antimony telluride thin films deposited by RF magnetron sputtering on flexible substrate using different. J. Electron. Mater..

[21] Toshima N (2015). Novel hybrid organic thermoelectric materials: three-component hybrid films consisting of a nanoparticle polymer complex, carbon nanotubes, and vinyl polymer. Adv. Mater..

[22] Hyland M, Hunter H, Liu J, Veety E, Vashaee D (2016). Wearable thermoelectric generators for human body heat harvesting. Appl. Energy.

[23] Guan M, Wang K, Xu D, Liao WH (2017). Design and experimental investigation of a low-voltage thermoelectric energy harvesting system for wireless sensor nodes. Energy Convers. Manag..

[24] Madan D (2013). High-performance dispenser printed MA p-type Bi_0.5_Sb_1.5_Te_3_ flexible thermoelectric generators for powering wireless sensor networks. ACS Appl. Mater. Interfaces.

[25] Dilhac J-M (2014). Implementation of thermoelectric generators in airliners for powering battery-free wireless sensor networks. J. Electron. Mater..

[26] Zhou C, Kong J, Yenilmez E, Dai H (2000). Modulated chemical doping of individual carbon nanotubes. Science.

[27] MacLeod BA (2017). Large n-and p-type thermoelectric power factors from doped semiconducting single-walled carbon nanotube thin films. Energy Environ. Sci..

[28] Nonoguchi Y (2013). Systematic conversion of single walled carbon nanotubes into n-type thermoelectric materials by molecular dopants. Sci. Rep..

[29] Horike S, Wei Q, Kirihara K, Mukaida M (2020). Water-processable n-type doping of carbon nanotubes via charge transfer with imidazolium chloride salt. Chem. Phys. Lett..

[30] Liu Y, Dai Q, Zhou Y, Mao X (2019). High-performance n-type carbon nanotubes composites: improved power factor by optimizing the acridine scaffold and tailoring the side chains. ACS Appl. Mater. Interfaces.

[31] Oshima K (2017). Improvement of stability of n-type super growth CNTs by hybridization with polymer for organic hybrid thermoelectrics. Synth. Met..

[32] Nakashima Y (2019). Air-stable n-type single-walled carbon nanotubes doped with benzimidazole derivatives for thermoelectric conversion and their air-stable mechanism. ACS Appl. Nano Mater..

[33] Nonoguchi Y (2016). Simple salt-coordinated n-type nanocarbon materials stable in air. Adv. Funct. Mater..

[34] Seki Y, Nagata K, Takashiri M (2020). Facile preparation of air-stable n-type thermoelectric single-wall carbon nanotube films with anionic surfactants. Sci. Rep..

[35] Giannozzi P (2009). Quantum espresso: a modular and open-source software project for quantum simulations of materials. J. Phys. Condens. Matter.

[36] Liu J (2017). The electronic properties of chiral carbon nanotubes. Comput. Mater. Sci..

[37] Yusupov K (2020). Enhancing the thermoelectric performance of single-walled carbon nanotube-conducting polymer nanocomposites. J. Alloys Compd..

[38] Maruyama, S. *Tables of Those Value Up to (40,40) Nanotubes*. http://www.photon.t.u-tokyo.ac.jp/~maruyama/kataura/chiraldata.html (2002).

[39] Seki Y, Takashiri M (2020). Freestanding bilayers of drop-cast single-walled carbon nanotubes and electropolymerized poly(3,4-ethylenedioxythiophene) for thermoelectric energy harvesting. Org. Electron..

[40] Frey, J. T. & Doren, D. J. TUBEGEN 3.4, University of Delaware, Newark, DE (2011).

[41] Grimme S, Antony J, Ehrlich S, Krieg H (2010). A consistent and accurate ab initio parametrization of density functional dispersion correction (DFT-D) for the 94 elements H-Pu. J. Chem. Phys..

[42] Perdew JP (1996). Generalized gradient approximation made simple. Phys. Rev. Lett..

[43] Kayang KW (2019). A comparative study of the interaction of nickel, titanium, palladium, and gold metals with single-walled carbon nanotubes: a DFT approach. Results Phys..

[44] Bandow S, Asaka S, Saito Y, Rao AM (1998). Effect of the growth temperature on the diameter distribution and chirality of single-wall carbon nanotubes. Phys. Rev. Lett..

[45] Pimenta MA (2007). Studying disorder in graphite-based systems by Raman spectroscopy. Phys. Chem..

[46] Murakami Y (2004). Growth of vertically aligned single-walled carbon nanotube films on quartz substrates and their optical anisotropy. Chem. Phys. Lett..

[47] Zhou Y (2019). Highly conducting, durable and large area carbon nanotube thick films for stretchable and flexible electrodes. Appl. Phys. Lett..

[48] Avery AD (2016). Tailored semiconducting carbon nanotube networks with enhanced thermoelectric properties. Nat. Energy.

[49] Hirano A (2016). Origin of the surfactant-dependent redox chemistry of single wall carbon nanotubes. ChemNanoMat.

